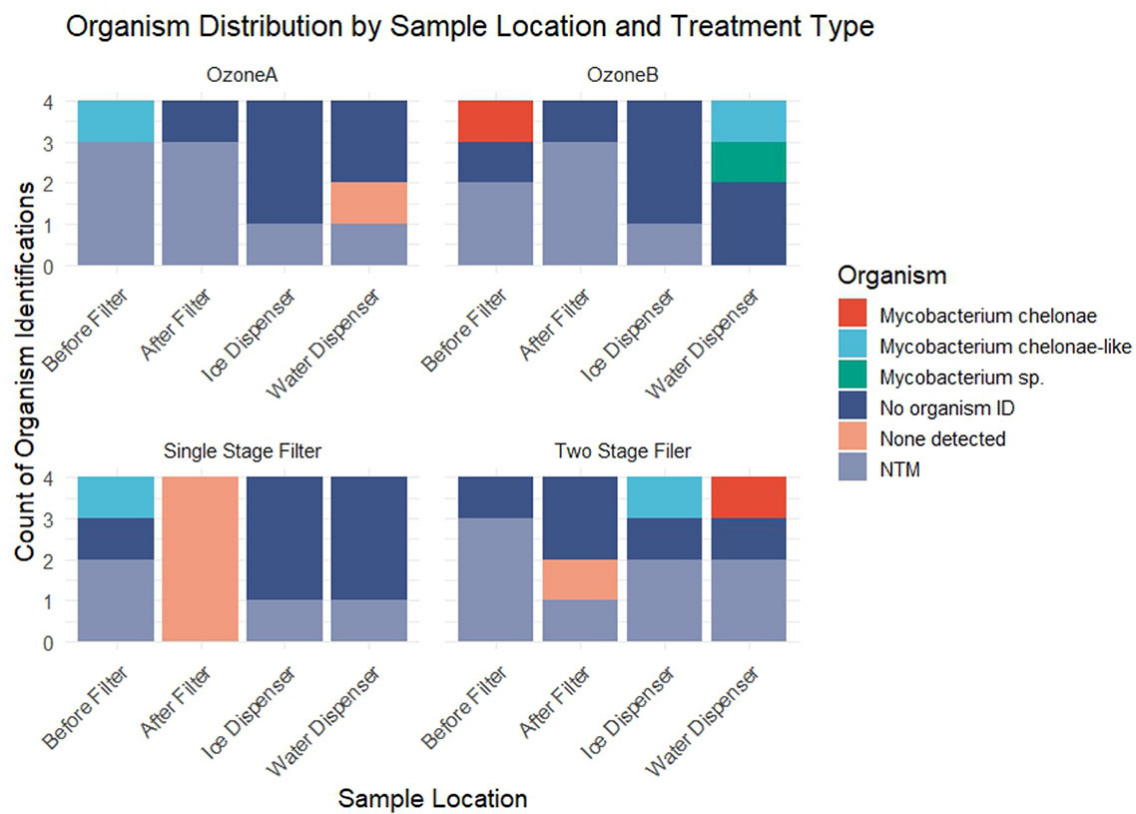# 29 Ventilator Associated Event (VAE) Reduction Utilizing Bundle Sets in the Neonatal Intensive Care Unit

**DOI:** 10.1017/ash.2026.10473

**Published:** 2026-06-23

**Authors:** Andrea Ankrum, Mike Torsell, Dave Strohmier, Michael Mattar, Katelynn Maguire, Felicia Scaggs Huang

**Affiliations:** 1 Cincinnati Childrens Hospital Medical Center; 2 CCHMC; 3 Water sampler; 4 Plant Engineering; 5 Cincinnati Children’s; 6 Cincinnati Children’s Hospital Medical Center

## Abstract

**Background:** Mycobacterium chelonae and other non-tuberculous mycobacteria (NTM) isolated from ice/water dispensers have been implicated in healthcare outbreaks. We present the results of mitigation activities utilized to reduce M. chelonae from ice/water dispensers as part of an outbreak response. **Methods:** Interventions were performed in phases. Phase 1 involved ensuring compliance with the manufacturer’s ice/water dispenser cleaning protocol for 11 dispensers involved in the outbreak. Phase 2 compared monthly, bi-monthly, and quarterly cleaning outcomes of seven dispensers. Phase 3 evaluated point source controls on four dispensers in a test environment, two with a 0.005 µm microbial filter either solo (single stage) or in combination with a particle filter (two stage) and two with an ozone treatment system (each with a particle filter). In all phases, monthly environmental culturing for NTM was performed from four sources (water before the filter, water after the filter, ice and water from the spout) on each dispenser for four months. Additionally, swab samples of the waterspouts were collected in Phase 1. Several incoming water sources were also assessed for NTM. Identification of NTM from culture was performed by matrix-assisted laser desorption/ionization-time of flight mass spectrometry. **Results:** Phase 1 revealed one NTM positive sample from an ice/water dispenser in the first month with an increasing number of positive results each subsequent month with 40 /44 samples with NTM including 10 M. chelonae in month four. Phase 2 results did not show a significant difference in NTM results between groups by cleaning frequency. Phase 3 results continued to show positive NTM across all dispensers with the single stage microbial filter outperforming the other point source controls (Figure 1). The hospital incoming water results confirmed the presence of M. chelonae along with other species of NTM. **Conclusions:** Recovery of NTM including M. chelonae from the incoming water supply highlights the known risk from municipal water. Upon implementation of point source controls, the single stage filter had no NTM recovered from water immediately after the filter which may be beneficial. Despite robust and increased cleaning frequency, NTM was still recovered from all dispensers which suggests the presence of biofilm. Improved cleaning protocols are needed from ice/water dispenser manufacturers to address NTM biofilm issues in healthcare settings. Additionally, national guidance on management of NTM in hospital water systems to reduce risk to vulnerable patient populations is overdue.

**Figure:**